# Left atrium dysfunction by CMR in aortic valve stenosis

**DOI:** 10.1186/1532-429X-17-S1-P6

**Published:** 2015-02-03

**Authors:** Morgane Evin, Alban Redheuil, Jerome Lamy, Carine Defrance, Elie Mousseaux, Philippe Cluzel, Karine Clément, David Rosenbaum, Nadjia Kachenoura

**Affiliations:** 1 Sorbonne Universités, UPMC Univ Paris 06, UMR 7371, UMR_S 1146, Laboratoire d'Imagerie Biomédicale, Paris, France; 2 ICAN | Institute of Cardiometabolism And Nutrition, Paris, France; 3 Cardiology Departement, European Hospital Georges Pompidou, Paris, France; 4 Departement de radiology - la Pitié Salpétriêre, Paris, France

## Background

Cardiac Magnetic resonance (CMR) imaging is able to characterize with high accuracy left atrial (LA) size and volumes as well as dense myocardial fibrosis. The addition of functional parameters such as LA myocardial strain would enhance CMR usefulness for a comprehensive LA characterization. Our aim was to evaluate the ability of strain values obtained by CMR to characterize LA functional alterations in the setting of aortic valve stenosis (AVS).

## Methods

We studied 10 elderly healthy subjects (64±6y.) and 20 patients with aortic valve stenosis (73±15y), effective aortic area: 0.67±0.36cm2 and mean gradient: 38.8±20.6mmHg) who underwent MRI exam on 1.5T magnet, including three SSFP long axis cine loops. A validated custom feature tracking algorithm was used on this data to calculate global and regional, longitudinal strain (Sl), longitudinal strain-rate (SRl), radial motion fraction (Mr) and radial relative velocity on standardized LA segments. These functional parameters were assessed for reservoir, conduit and LA contraction phases. LA volumes were calculated using the Qmass software.

## Results

LA volumes and ejection fraction were significantly lower in AVS (p<0.05). For all LA phases, longitudinal strain values were significantly (p<0.04) reduced in AVS patients (reservoir phase : 22.6±4.9% for controls and 12.4±6.5% for AVS; conduit phase :8.7±3.1% for controls and 2.7±1.8% for AVS and LA contraction phase: 14.0±4.1% for controls and 9.8±5.9% for AVS). Same trend was observed for radial measurements with Mr_R_=26.8±5.5%Mr_E_=11.6±2.7% and Mr_A_=15.3±4.1% being higher for controls than AVS patients Mr_R_=13.8±7.4%, Mr_E_=3.3±2.3% and Mr_A_=10.4±6.9% (p<0.01). Longitudinal strain rates and radial relative velocities were also significantly reduced in AVS (p<0.02) for reservoir, conduit and LA contractionphases. Longitudinal reservoir strain to LA contraction ratio (1.4±0.4 vs. 1.7±0.3, p=0.01) and radial motion fraction ratio (1.8±0.2 vs. 1.5±0.8, p=0.002) were significantly lower in AVS. Regarding strain rate and relative velocity, only radial relative velocity E' to A' ratio was significantly reduced in AVS (0.7±0.3 vs. 0.5±0.3, p=0.02).

## Conclusions

Conventional and standardized cine SSFP images analyzed with feature tracking resulted in LA functional parameters which fully described atrial myocardial function with its longitudinal and radial components and its reservoir, conduit and LA contraction phases. Such parameters were able to characterize LA functional impairment secondary to AVS. The addition of the proposed feature tracking tool might enhance the diagnostic value of routine LA assessment in CMR.

## Funding

N/A.

